# Unveiling the enigma of the blood–brain barrier in glioblastoma: current advances from preclinical and clinical studies

**DOI:** 10.1097/CCO.0000000000000990

**Published:** 2023-09-07

**Authors:** Mohammed H. Ahmed, Michael Canney, Alexandre Carpentier, Maya Thanou, Ahmed Idbaih

**Affiliations:** a School of Cancer & Pharmaceutical Sciences, King's College London, London, UK; b Carthera, Lyon; c Sorbonne Université, AP-HP, Institut du Cerveau - Paris Brain Institute - ICM, Inserm, CNRS, Hôpitaux Universitaires La Pitié Salpêtrière - Charles Foix, Service de Neurochirurgie; d Sorbonne Université, AP-HP, Institut du Cerveau - Paris Brain Institute - ICM, Inserm, CNRS, Hôpitaux Universitaires La Pitié Salpêtrière - Charles Foix, DMU Neurosciences, Service de Neurologie 2-Mazarin, Paris, France

**Keywords:** blood–brain barrier, brain tumors, glioblastoma, local drug delivery

## Abstract

**Purpose of review:**

Glioblastoma (GBM), the most prevalent primary brain malignancy in adults, poses significant challenges in terms of treatment. Current therapeutic strategies for GBM patients involve maximal safe resection, followed by radiotherapy with concurrent and adjuvant temozolomide. However, despite this multimodal approach for GBM, the prognosis of GBM patients remains dismal because of their inherent primary and secondary resistances to treatments.

**Recent findings:**

Several molecular and cellular mechanisms, including the presence of the blood–brain barrier (BBB), contribute to these resistances. The BBB, comprising multiple layers surrounding brain vessels, acts as a barrier limiting effective drug delivery to the brain. Invasive and noninvasive tools to deliver drugs and pharmaceutical formulations locally or systemically are continuously evolving to overcome the BBB in GBM toward improving drug bioavailability in the brain and reducing systemic toxicities.

**Summary:**

Preliminary studies utilizing these approaches have demonstrated promising results in terms of safety and signals of efficacy during early-phase clinical trials. However, further work through additional clinical trials is necessary to evaluate the potential clinical benefits for GBM patients.

## INTRODUCTION

Despite the advances in molecular and genetic understanding of glioblastoma (GBM) since 2005, there have been no clinically approved alternatives for Temozolomide (TMZ) as the primary treatment for the entire population of GBM patients. The addition of lomustine or tumor-treating fields (TTF) to the standard of care has demonstrated clinical benefit in sub-populations of GBM patients [1,2]. The blood–brain barrier (BBB) is a biophysical and biochemical specialized structure that is exclusive to the central nervous system (CNS) blood vessels. The BBB acts as a defensive barrier shielding the brain from harmful molecules circulating in the bloodstream while ensuring the adequate supply of nutrients and hydro electrolytic compounds required to maintain homeostasis. The changes in the microenvironment of GBM and the process of angiogenesis are partially responsible for the development of irregular, disorganized, large, and permeable micro-vessels, which contribute to the functional alteration of the blood–tumor barrier (BTB). Despite the BBB's functional changes in the tumor, it remains partially intact. As a result, the penetration of chemotherapy agents is enhanced but not to the extent observed in the complete absence of the BBB. Furthermore, tumor cells located outside the region of BBB alteration (i.e. the bed around the tumor) remain protected. 

**Box 1:**
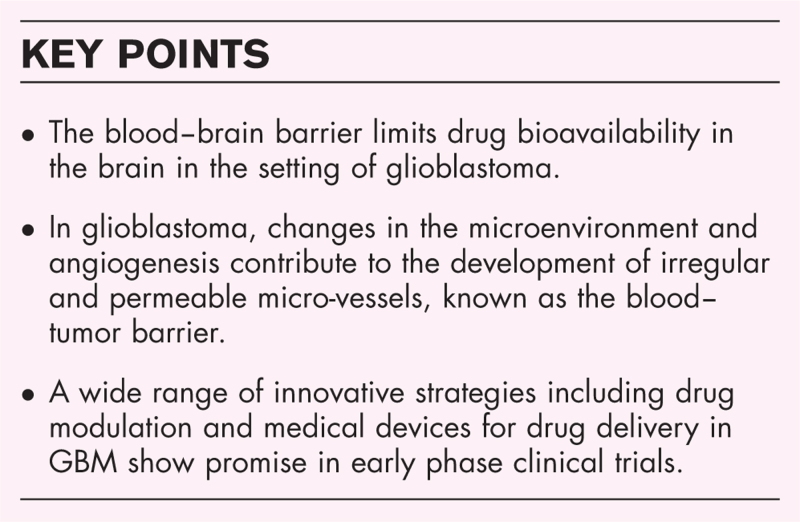
no caption available

## GLIOMA-ASSOCIATED NEOVASCULARIZATION

**FIGURE 1:**
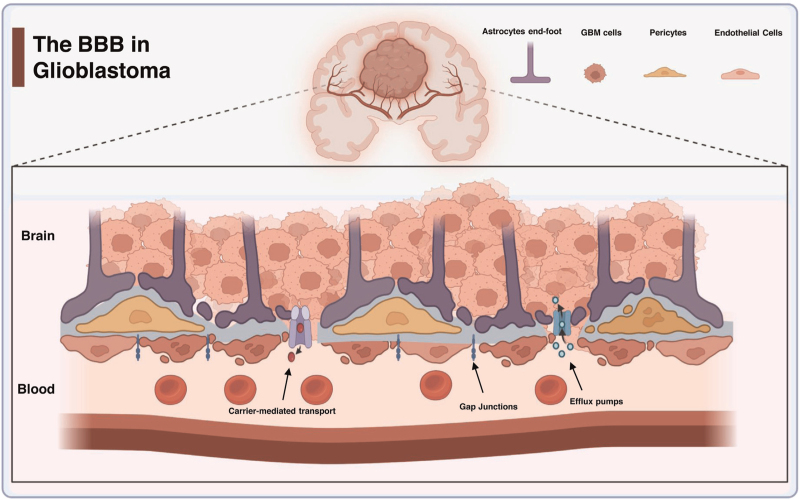
Representative illustration of the major changes in blood–brain barrier in glioblastoma. These abnormalities can be noticed as endothelial cell hyperplasia, higher vascular permeability, aberrant pericyte distribution, loss of astrocytes’ end-feet, and tortuous vasculature which modulate the physiological functions of transcellular passive transport, paracellular transport, carrier-mediated transports, and efflux pumps mechanisms. Efflux pump transport mechanisms (ABCB1, ABCC1, ABCG2) [31^▪^] are overexpressed in GBM and expel drugs to the blood following their transport to the brain parenchyma. GBM, glioblastoma.

The BBB is a biochemical and biophysical barrier between the cerebral vascular cell's lumen and the brain parenchyma, and it is composed of endothelial cells, pericytes, and astrocytes end-feet. Endothelial cells are the main cellular component of the BBB; they are joined together by tight junctions preventing the paracellular pathways while pericytes are a type of vascular cells that surrounds the endothelial cells. They maintain the integrity of the BBB and regulate blood flow to the brain. On the other hand, astrocytes are glial cells that surround the blood vessels in the brain. In GBM, the disruption of the BBB can be attributed to several factors: GBM cells directly invading blood vessels and disrupting endothelial cells, dysregulation of signaling pathways that can cause dysfunction in BBB organization, activation of inflammatory processes that increase vascular permeability, and the process of tumor neoangiogenesis. Glioma-associated neovascularization is a complex and regulated process and is highly dependent on the balance between separate pathways and consequently participates in the deformity of the BBB in GBM [3^▪^]. Figure 1 represents the structural abnormalities that have been observed in the BBB in GBM.

## BLOOD–BRAIN BARRIER AS A LIMITATION FOR THERAPEUTIC AGENTS

Large therapeutic agents face challenges in crossing the BBB; however, small molecules have a higher likelihood, with approximately 20% of them capable of crossing the BBB. In the case of the disrupted BBB, oral administration of TMZ enables 20–30% of the drug to cross the BBB, while nitrosoureas and platinum derivatives have a lower probability of reaching the brain [4^▪^,5^▪^]. Over the past few years, there has been a growing need for innovative strategies to address and overcome the limitations in delivering such compounds to the brain. These strategies involve both local and systemic administration of free drugs or alternative drug formulations, along with techniques aimed at disrupting the BBB to enhance drug delivery or modify drug formulations for better bioavailability [6^▪^].

## CONVECTION-ENHANCED DELIVERY

**FIGURE 2:**
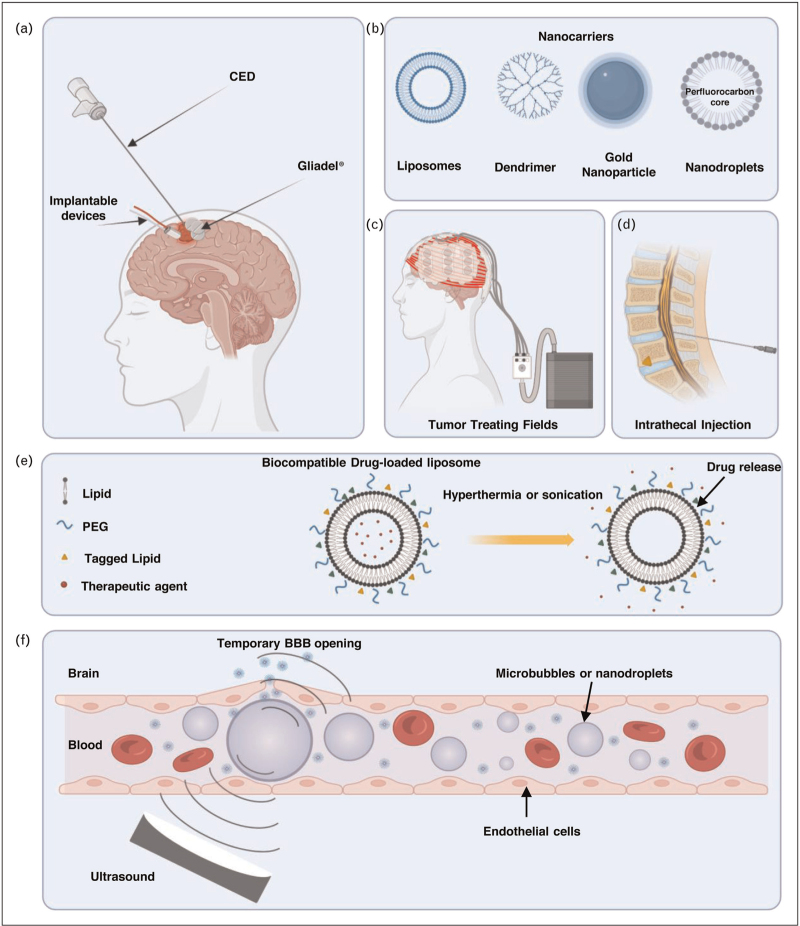
Representative summary of the current drug delivery techniques used to overcome the blood–brain barrier in glioblastoma.

Convection-enhanced delivery (CED) is based on the insertion of a catheter for the direct administration of drugs to the targeted brain region/tumor volume. The catheter is placed with a positive pressure pump that allows the drug to be delivered slowly over a specific time (Fig. 2a). Several antineoplastic agents were tested using CED (e.g. cisplatin, methotrexate, paclitaxel, nimustine, topotecan, and carboplatin). The success of CED is highly dependent on the chemotherapeutic agent, the tumor location, and the surgical experience. In a recent phase 2 clinical trial, 44 patients received a single injection of genetically engineered interleukin-4 (MDNA55, Medicenna, Toronto, Canada). No significant adverse effect was correlated to direct MDNA55 injection and patients who received the MDNA55 survived longer with median overall survival of 12.4 months compared with 7.7 months in the synthetic control group [7]. Although the invasive characteristics of CED and the substantially associated neurotoxicity pose constraints that necessitate the careful monitoring of neurological adverse effects, this method is still being used to test novel therapeutic strategies. A recently announced clinical study is ongoing to evaluate the efficacy of CED of MTX-110 – soluble panobinostat – in recurrent GBM patients (NCT05324501).

## BIODEGRADABLE WAFERS

A drug-loaded polymer wafer (Gliadel) was developed and approved by the Food and Drug Administration (FDA) in 1996 and used for the direct delivery of carmustine to brain tumors. A recently published meta-analysis studied the overall survival and progression-free survival of newly diagnosed high-grade glioma patients treated with the standard of care plus Gliadel wafers. Twelve trials were identified and eight were excluded for a few reasons, that is, recurrent GBM, no standard-of-care arm, and not including a noncarmustine wafer treatment group for comparison. Only four clinical trials were included in the analysis. Although the study exhibits several limitations, it revealed that carmustine wafers show a significant role in improving the overall survival of newly diagnosed GBM patients [8^▪^]. There are several constraints associated with this approach. The initial limitation is known as the sink effect, characterized by a rapid decrease in drug concentration following its release from the polymer. The second constraint relates to the rheological properties of the Gliadel formulation, which contribute to elevated local toxicities. Furthermore, infection and development of hydrocephalus were reported. Scientific labs are actively exploring soft and malleable formulations in mouse models of GBM to address these limitations, with the aim of eventually conducting clinical trials on these improved formulations [9^▪^].

## DIRECT INTRATUMOR INJECTIONS

Local delivery of a free drug solution or a formulation under MRI control allows a proper delivery protocol. The main advantage of intratumoral local delivery is to avoid the systemic toxicity of chemotherapies. Numerous chemotherapies can be delivered by this method. However, some adverse effects such as neurotoxicity might be a limitation, that is, taxanes and platinum derivatives may induce seizures that limit their usage through this route [10].

A single-arm, phase 1, multicenter clinical trial to evaluate the safety and efficacy of polymer-based cisplatin-loaded gel (TumoCure) gel formulation is currently ongoing. Although the study evaluates the drug safety and efficacy in head and neck tumors, which is not limited to GBM patients, it might open a new possibility in the foreseen years (NCT05200650). A novel formulation of mechanically matching the rheological properties of brain tissue, biodegradable gel formulation increased survival in preclinical models of GBM. [9^▪^].

## IMPLANTABLE MICRODEVICES

To prevent toxicities associated with free drug intratumoral injections, microdevices are currently under preclinical [11^▪^] and clinical testing (NCT04135807) to deliver chemotherapeutic agents. In this first-in-human clinical trial, six patients were enrolled with either general anesthesia (4/6) or conscious sedation (2/6). The device is designed to release small doses of drugs. Up to two microdevices were implanted per patient and all microdevices were retrieved following the drug administration (Fig. 2a). The insertion of the microdevices did not induce significant surgical complications compared with the control group. Doxorubicin and lapatinib were used in this study as they can be detected through fluorescent analysis. Molecular analysis of DNA damage was performed and high variation –9.8% in some patients and up to 65% in other patients – in DNA damage was observed. Although this is the first report discussing the implantable microdevices in GBM, a larger clinical trial should be warranted to allow the expansion of such a novel technique [12^▪^]. It is worth mentioning that the brain tissue could react to foreign body implantation through the activation of inflammatory and fibrotic cascades, therefore, further development of biocompatible implants will require more in-depth analysis of the impact of such technologies in preclinical and clinical trials [13].

## DIRECT INTRATHECAL INJECTION

The limited distribution of cerebrospinal fluid (CSF) injections to the brain parenchyma limited the use of this method for GBM. Methotrexate and cytarabine show an acceptable safety profile when administered intrathecally; however, vincristine is contraindicated for direct intrathecal injection (Fig. 2d). Based on the data collected over 7 years in a recently published retrospective study, an encouraging median overall survival of 27.8 months among GBM patients with leptomeningeal dissemination who received intrathecal methotrexate injection with systemic chemotherapy [14].

## DRUG MODULATION STRATEGIES USING NANOCARRIERS

The primary goal of nanocarriers such as liposomes, nanodroplets, and dendrimers (Fig. 2b) is to improve the efficacy and safety of therapeutic agents. Encapsulating drugs or other bioactive molecules within their structure, nanocarriers protect them from degradation, enhance their stability, and control their release at the specific site [15]. Current treatment schemes with chemotherapy have immediate and substantial adverse effects for cancer patients, including hair loss, digestive problems, bone marrow suppression, nausea, numbness, and general weakness. Encapsulating therapeutic agents within inert liposomes reduces the deleterious effects of chemotherapy on healthy tissues. Ultrasound and/or hyperthermia-activated liposomes are usually achieved through appropriate molar concentrations of the lipids used in their formulations. Thermosensitive liposomes melt and release the encapsulated drugs when the physiological temperature is higher than 42 ^o^C. This strategy is currently under preclinical evaluation in GBM mouse models [16]. In preclinical mouse models of GBM sonosensitive liposomes increased doxorubicin accumulation in the brain following ultrasound-mediated drug delivery (Fig. 2e) [17^▪^].

Nanodroplets were first identified in 1998 as formulations that respond to ultrasound. However, their initial use as carriers for drugs was reported in 2007. In 2013, nanodroplets were combined with ultrasound-mediated BBB opening (UMBO) for a specific purpose. The composition of nanodroplets includes a lipid shell and a core that must be bio-inert, hydrophobic, and capable of safely traveling through the circulatory system until it reaches a gaseous state. Therefore, the core material needs to have an appropriate boiling point. Unlike microbubbles, which often use air, nitrogen, or sulfur hexafluoride as their core, nanodroplets utilize perfluorocarbon to meet these specific requirements. Nanodroplets are considered a valuable tool for delivering hydrophobic agents in ultrasound-mediated drug delivery [18^▪^].

Dendrimers are another type of nanocarrier, they are highly defined, artificial, hyperbranched tree-like structure, which can be developed as small as 20 nm in size. The relatively smaller size of dendrimers as a nanocarrier and their high loading capacity makes them one of the preferable tools for drug delivery [19^▪^]. Not to mention, ultrasmall theranostic gadolinium-based nanoparticle (AGuIX, Lyon, France) is currently under clinical evaluation (NCT03818386) [20]. Therefore, a proper selection of type, size, physiochemical properties, and bioavailability of the nanocarrier allows them to be effective tools in future clinical studies [21].

## TUMOR TREATING FIELDS

Tumor treating fields (TTF) is one of the most recent FDA-approved noninvasive therapeutic strategies to treat GBM. Low-intensity TTFs with immediate frequency (100–300 kHz) interfere with GBM cell proliferation by interfering with mitotic processes. Previous studies have shown that TTFs impair microtubule polymerization and septin filaments, which are required during mitosis for proper chromosome segregation and cytokinesis [22]. TTFs application against solid tumors has been investigated; however, the effects on the immune cell population in the tumor microenvironment are still unclear. A recent report has investigated the possibility of TTFs at these frequencies disrupting the BBB barrier [23^▪^]. The exact mechanism is still not fully understood; however, it has been investigated that TTFs alter the junctional proteins claudin-5 and ZO-1 at the BBB allowing a reversible BBB disruption. Further studies are required to fully understand the potential molecular effects of TTFs (Fig. 2c) [24^▪^].

## ULTRASOUND-MEDIATED BLOOD-BRAIN BARRIER OPENING

Focused or unfocused ultrasound in combination with bubble-forming nuclei (such as nanobubbles/microbubbles and nanodroplets) represents an innovative and noninvasive solution for permeating the BBB and has been used in a variety of clinical applications since the 1950s (Fig. 2f). When subjected to ultrasound-mediated blood-brain barrier opening (FUS), bubble-forming nuclei expand and collapse in a process called cavitation, generating highly localized mechanical forces that help to improve the permeation of therapeutics. UMBO was used in preclinical models to bypass BBB efflux transporters and increase the brain's penetration of a wide variety of therapeutics. Low-intensity pulsed ultrasound can be delivered to the brain to induce a safe oscillation of intravenously injected microbubbles within blood vessels. Oscillation of these microbubbles opens the BBB by reversibly disrupting the tight junctions between endothelial cells [6^▪^,18^▪^,^25^]. A range of drugs have been tested for use with UMBO for treating gliomas and include temozolomide, carmustine, irinotecan, nab-paclitaxel, aPD-1, carboplatin, doxorubicin, and drug-loaded liposomes [5^▪^,6^▪^]. In preclinical models of GBM, focused ultrasound increased the delivery of chemotherapeutic agents [26] antibodies [27^▪^,^28^] and immunotherapeutic agents [29]. NaviFus (New Taipei, Taiwan) is currently evaluating the efficacy of focused ultrasound on antibody delivery in clinical trials (NCT04446416). Another recently published phase 1 clinical trial utilized an implantable low-intensity pulsed ultrasound system (SonoCloud-9, Carthera, Lyon, France) to overcome the BBB in recurrent GBM patients and successfully enhanced the concentration of carboplatin by 5.9-fold compared with nonsonicated brain in GBM patients [30^▪▪^].

## CONCLUSION

Invasive brain tumors are resistant to treatments because of multiple factors including the presence of the BBB. Overcoming the BBB can improve drug bioavailability and efficacy against brain cancers. It can also reduce systemic toxicities. Promising strategies are being investigated in early clinical trials, but further comparative trials are necessary for robust evaluation.

## Acknowledgements

*The authors would like to acknowledge the generous Grant Award 2022LPTINN2S12 from Children's Cancer and Leukaemia Group and NCA-DGOS-Inserm_12560 SiRIC CURAMUS by the French National Cancer Institute, the French Ministry of Solidarity and Health and Inserm. The authors would like to acknowledge the use of Biorender to draw figures under agreement numbers DK25HF227P and RT25HF6LRX for the institution license.*

### Financial support and sponsorship

*None.*

### Conflicts of interest

*M.T. is the founder of Apeikon Therapeutics. A.I. reports research grants and others from Carthera, Transgene, Sanofi, Nutritheragene; travel fundings from Enterome and Carthera; personal fees and other from Leo Pharma, Novocure, Novartis, Boehringer Ingelheim. M.C. is an employee of Carthera and has patents and stock options related to the technology presented. A.C. is paid as consultant for Carthera and has ownership interest in Carthera. M.C. is an employee of Carthera.*

## REFERENCES AND RECOMMENDED READING

Papers of particular interest, published within the annual period of review, have been highlighted as:

▪ of special interest
▪▪ of outstanding interest
